# The League of Mercy and Gifts in Kind

**Published:** 1921-03-26

**Authors:** 


### THE LEAGUE OF MERCY AND GIFTS IN KIND.

For many years we have had a column devoted to gifts and bequests, and it is remarkable that gifts in kind were rarely or never included among them. The reason was that these gifts were limited to Pound Days and occasional presents. They were neither greatly sought nor deliberately organised, so were mentioned separately, when mentioned at all. The war, however, encouraged the regular pursuit of gifts in kind, which hitherto only a few progressive hospitals, like Addenbrooke's at Cambridge, had really tried to make an important part of their receipts. The ultimate consequence has been to promote gifts in kind to special journalistic mention. They have had their revenge and overleaped the Gifts and Bequests column. A good instance of the value which they now possess in the minds of institutional officers comes from Exeter, where the Royal Devon and Exeter Hospital boasts both an egg week and a potato week. These efforts produced last year 25,000 eggs, representing (we presume) a value of at least £300 in money; and fourteen tons of potatoes.

Now, once again, we venture to suggest that the Provincial Hospitals have given a lead to London hospitals in this matter. It is true that only those hospitals on the fringe of the Metropolis have market gardens or farmers close at hand. But, from the London point of view, Covent Garden should be worth a hundred farms, and the retailers who come to market there, many potato weeks and broad acres. We therefore venture to suggest that either one of the great Funds, perhaps the League of Mercy, which is particularly concerned with the small giver, or separate hospitals, should get into regular touch with Covent Garden, and through, or simultaneously with, this attempt approach their local greengrocers and dairymen for regular contributions of their produce. It is money with which most people are loath to part. Even a miser will part with his old chest sooner than with a fraction of its worth in cash. A tradesman, whose vocation leads him to expect to receive money from others, while it encourages him to part as quickly as possible with his goods, by parity of reasoning, will think less of presenting a sack of potatoes than of taking a hospital donation of half its value from the till. Consequently gifts in kind are the gifts to seek from tradesmen.

These gifts should, preferably, not be sought by hospital secretaries through letters, for your tradesman is rarely a sympathetic correspondent. They should be sought by personal interview; and the interviewer, if possible, should be a neighbouring resident (though not necessarily a customer), and, best of all, a female one. Where is such a person to be found? Obviously in the ranks of the ladies affiliated to the local centres of the League of Mercy. The Secretary of the League, Miss A. Eva Milnes, or Sir James Harrison, or Sir William Collins, should have their quiver full of such active women.

There is another reason why the policy of organising such gifts in kind deserves the attention of the League. The reason is this. The League of Mercy has now passed its adolescent stage. It is successfully established. In other words, the original impulse has borne its fruit; and, in consequence, if the impulse is not to stultify, a new channel for its efforts should be found. Such a channel is offered by a policy of collecting, beside small donations in cash, gifts in kind through the League's local women workers in London. Everyone wearies at last of collecting money by personal visits. But there is not the same reluctance to solicit gifts of those material goods with which the object of the visit is accustomed to part. We therefore commend this policy to the sympathetic consideration of the secretary of the League of Mercy to diversify her work and in order that the League may do its appropriate share side by side with the other great Funds in the present joint effort to finance by wider means the London hospitals.

